# A Pocket Text-Book of Chemistry and Physics

**Published:** 1900-11

**Authors:** 


A POCKET TEXT-BOOK OF CHEMISTRY AND PHYSICS. By WALTON MARTIN, M. D., and WILLIAM H ROCKWELL, JR., A. B., M D., of the College of Physicians and Surgeons, New York. In one 12mo volume of 366 pages, with 137 illustrations. Philadelphia and New York: Lea Brothers & Co. [Price, cloth, $1.50; flexible red leather, $2.00.]

Half of this volume is devoted to physics. While the section on chemistry is excellent as far as it goes, it is altogether too brief to be of much service either to physician or student, and physiological chemistry is practically omitted. One regrets that the entire book had not been given over to chemistry, or that physics had been given less space. M. J. F.

